# Comparative effectiveness of East Asian traditional medicine for treatment of idiopathic short stature in children: A protocol for systematic review and network meta-analysis

**DOI:** 10.1097/MD.0000000000022856

**Published:** 2020-10-23

**Authors:** Boram Lee, Chan-Young Kwon, Soobin Jang

**Affiliations:** a Clinical Medicine Division, Korea Institute of Oriental Medicine, Yuseong-gu, Daejeon; b Department of Oriental Neuropsychiatry, Dong-eui University College of Korean Medicine, Busanjin-gu, Busan, Republic of Korea.

**Keywords:** East Asian traditional medicine, idiopathic short stature, network meta-analysis, study protocol, systematic review

## Abstract

**Background:**

There are many East Asian traditional medicine (EATM) therapies that are widely used and effective for idiopathic short stature (ISS) in children. However, the comparative effectiveness of these therapies remains unclear. We describe the methods that will be used to comparatively evaluate the efficacy and safety of EATM therapies for the treatment of pediatric ISS.

**Methods and analysis:**

Fourteen electronic English, Korean, Chinese, and Japanese databases will be searched up to August 2020 for relevant randomized controlled trials of various EATMs for the treatment of pediatric ISS, without language or publication status restrictions. The primary outcome will be growth-related anthropometric indicators, and acceptability, measured through drop-outs that occur during treatment for any reason. We will conduct a pairwise meta-analysis for direct comparisons if multiple studies use the same types of intervention, comparison, and outcome measure. A frequentist network meta-analysis will be performed to summarize the available direct and indirect evidence regarding various EATM options for pediatric ISS. The risk of bias for the included studies will be evaluated using the Cochrane Collaboration's risk of bias tool.

**Conclusions:**

The findings of this review will provide evidence for the comparative effectiveness and ranks of current EATMs and help to inform clinical practitioners, patients, and policy makers in decision making.

**Ethics and dissemination:**

Ethical approval is not required because individual patient data are not included. The findings of this systematic review will be disseminated through a peer-reviewed publication or conference presentations.

**Protocol registration number:**

OSF (URL: https://osf.io/s4vp7), PROSPERO CRD42020187160

## 1 Introduction

Idiopathic short stature (ISS) is a condition in which the individual's height is 2 standard deviations (SDs) or more below the corresponding average height for a given age, sex, and population group, in the absence of any systemic, endocrine, nutritional, or chromosomal abnormality.^[1]^ It is estimated that approximately 80% of children with short stature have ISS.^[2]^ ISS is a heterogeneous group with a wide variety of causes^[2]^ and there is no gold standard treatment for the condition. In July 2003, the US Food and Drug Administration approved the use of growth hormones (GHs) for ISS treatment^[3]^; however, the treatment is still controversial because of variable efficacy and high costs.^[4,5]^ Consequently, a guideline was released recommending against the use of GHs for the treatment of ISS.^[6]^

East Asian traditional medicines (EATMs) such as acupuncture, herbal medicine, and moxibustion are some of the most popular complementary and alternative medicines in the world and have been widely used for the treatment and prevention of various diseases for thousands of years. According to a cross-national survey conducted in East Asian countries in 2010, 34.4% of respondents in China, 26.5% in Japan, and 44.1% in Korea used EATMs in the past year.^[7]^ In a retrospective study analyzing the chief complaints of patients who visited the department of Korean Pediatrics in a Korean Medicine Hospital in the Republic of Korea for the first time, growth disorder was the most frequent chief complaint among school-aged children and adolescents and the number of patients that visited the hospital with this complaint has increased every year.^[8]^ Many experimental and clinical studies have shown that various EATM therapies have potential benefits for the treatment of ISS.^[9–11]^ However, comparative studies on the effectiveness of various EATM therapies have not been conducted thus far and the lack of evidence from direct comparisons between EATMs makes it difficult for clinicians to find the most effective therapies in clinical settings.

Network meta-analysis (NMA) can be used to assess the comparative effectiveness of multiple treatment strategies by evaluating direct and indirect evidence.^[12]^ In addition, it can provide the ranks of various treatment modalities according to their effectiveness, which can be useful for decision making in clinical settings.^[12]^

Therefore, we plan to conduct a systematic review and NMA to comprehensively summarize and evaluate the comparative effectiveness and safety of EATM therapies for ISS treatment in children, with the hope of providing worthwhile information on the best EATMs for health-care practitioners, patients, and policy makers.

## 2 Methods

### 2.1 Protocol registration

This protocol has been registered on the OSF registries (URL: https://osf.io/s4vp7) and PROSPERO platform (registration number: CRD42020187160). We will conduct a systematic review according to this protocol, but if protocol amendments occur, the dates, changes, and rationales for each amendment will be tracked in PROSPERO. This protocol is reported in accordance with the Preferred Reporting Items for Systematic Review and Meta-Analysis Protocols.^[13]^

### 2.2 Ethics and dissemination

Ethical approval will not be needed because the data used in this systematic review will not be individual patient data and there will be no concerns regarding privacy. The results will be disseminated through the publication of a manuscript in a peer-reviewed journal or presentation at a relevant conference.

### 2.3 Data sources and search strategy

One researcher (BL) will search Medline via PubMed, EMBASE via Elsevier, the Cochrane Central Register of Controlled Trials, the Allied and Complementary Medicine Database via EBSCO, and the Cumulative Index to Nursing and Allied Health Literature via EBSCO. We will also search the local databases in the Republic of Korea (Oriental Medicine Advanced Searching Integrated System, Korean studies Information Service System, Research Information Service System, Korean Medical Database, and Korea Citation Index), China (China National Knowledge Infrastructure, Wanfang data, and VIP), and Japan (CiNii). Each database will be searched for articles published until August 2020. We will search the reference lists of included studies and relevant review articles, as well as trial registries such as Clinicaltrials.gov. We will include not only the articles published in journals but also “gray literature” such as theses and conference proceedings. There will be no language restrictions. We will collect literature as comprehensively as possible through consultations with experts in pediatrics in Korean medicine. The search strategy for Medline is shown in Table 1 and will be modified and applied similarly for the other databases.

**Table 1:**
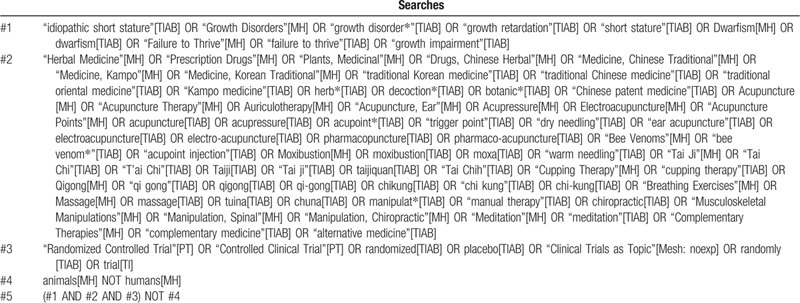
Search strategies for Medline.

### 2.4 Eligibility criteria

#### 2.4.1 Study design

We will only include parallel designed randomized controlled trials (RCTs). Studies that use inappropriate random sequence generation methods such as alternate allocation or allocation by birthdate will be excluded because they are quasi-RCTs, not RCTs. We will exclude cluster-randomized trials and cross-over trials to avoid possible sources of heterogeneity. In addition, articles for which only the abstracts are available such as conference proceedings will be excluded.

#### 2.4.2 Participants

We will only include RCTs targeting children with ISS based on the following diagnostic criteria: a height more than 2 SDs below the corresponding average height for a given age, sex, and population. RCTs targeting adults or children with other diseases that can cause short stature such as GH deficiency, Turner syndrome, and small for gestational age will be excluded. There will be no restrictions on the sex or race of the participants.

#### 2.4.3 Interventions and comparators

As treatment interventions, EATM therapies including acupuncture, herbal medicine, moxibustion, cupping therapy, qigong, Tai Chi, pharmacopuncture, bee venom, chuna, meditation, and a combination of these therapies will be included.

Comparators will be active controls including GH, placebo, no treatment, or the treatments listed as the interventions. For combined treatment groups, there will be no limits on the number of combined interventions for the intervention and comparator groups used since multiple interventions are used in actual clinical settings (eg, acupuncture plus herbal medicine plus chuna, herbal medicine plus GH). In multi-arm trials, study groups assessing interventions other than those mentioned above will not be eligible. Studies comparing different forms of the same EATMs such as those comparing herbal medicine A and herbal medicine B will be excluded.

#### 2.4.4 Outcomes

The primary outcome measures are as follows:

(1) Post-treatment value or changes after treatment in growth-related anthropometric indicators, such as height, adult height, predicted adult height, growth velocity, and height SD score
(2) Acceptability, measured through drop-outs that occur during treatment for any reason

The secondary outcome measures are as follows:

(1) Post-treatment value or changes after treatment in growth-related hormones such as GH, insulin-like growth factor-1, and insulin-like growth factor binding protein-3
(2) Drop-outs that occur during treatment because of any adverse event
(3) Adverse events that occur during treatment, measured based on the incidence

#### 2.4.5 Others

The most comprehensive report will be used if duplicates of the same study were published in more than 1 journal.

### 2.5 Study selection and data extraction

**Figure 1:**
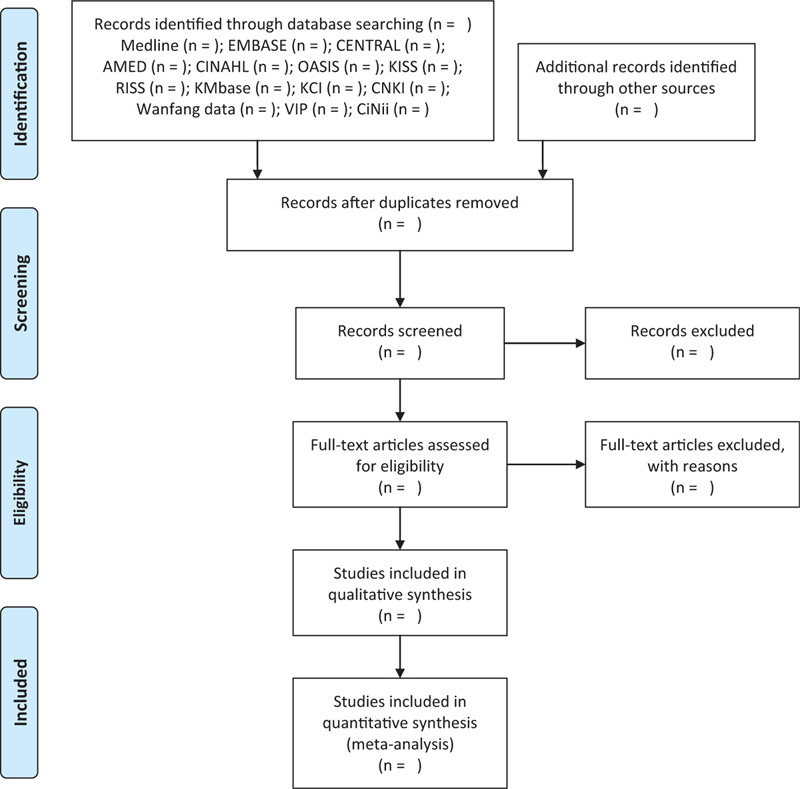
A PRISMA flow diagram of the literature screening and selection processes. AMED = Allied and Complementary Medicine Database, CENTRAL = Cochrane Central Register of Controlled Trials, CINAHL = Cumulative Index to Nursing and Allied Health Literature, CNKI = China National Knowledge Infrastructure, KCI = Korea Citation Index, KISS = Korean Studies Information Service System, KMbase = Korean Medical Database, OASIS = Oriental Medicine Advanced Searching Integrated System, RISS = Research Information Service System.

All searched articles from the databases and other sources will be imported into EndNote X8 (Clarivate Analytics, Philadelphia, PA), a reference management software program. After removing duplicates, we will evaluate the titles and abstracts of the searched studies for relevance and then evaluate the full texts of the remaining studies for final inclusion. The literature selection process will be reported in accordance with the Preferred Reporting Items for Systematic Review and Meta-Analysis flow diagram (Fig. 1). Using a standardized and pre-designed data collection form in Excel 2016 (Microsoft, Redmond), the following items will be extracted: the first author's name; year of publication; country; sample size, and number of dropouts; details about the participants, treatment interventions, and control interventions; duration of the intervention; outcome measures; results; and adverse events.

The study selection and data extraction will be conducted by 2 researchers (B Lee and CY Kwon) independently and discrepancies will be resolved through discussions with another researcher (S Jang). We will contact the corresponding authors of the included studies via e-mail to request additional information if the data are insufficient or ambiguous.

### 2.6 Risk of bias assessment

The risk of bias for the included studies will be assessed using the Cochrane Collaboration's risk of bias tool.^[14]^ We will assess random sequence generation, allocation concealment, blinding of participants and personnel, blinding of outcome assessments, incomplete outcome data, selective reporting, and other biases for each included study. Each domain will be rated as 1 of 3 levels: “low risk,” “unclear,” or “high risk.” The results of the evaluation will be recorded in Excel 2016 (Microsoft, Redmond, USA) and will be presented in a full review using Review Manager version 5.3 software (Cochrane, London, UK). Two independent researchers (B Lee and CY Kwon) will assess the risk of bias for the included studies and discrepancies will be resolved through discussions with another researcher (S Jang).

### 2.7 Data synthesis and analysis

#### 2.7.1 Pair-wise meta-analysis

Descriptive analyses of the details of the participants, interventions, and outcomes will be conducted for all included studies. We will conduct pairwise meta-analysis for direct comparisons if multiple studies employ the same types of intervention, comparison, and outcome measure using Review Manager version 5.3 software (Cochrane, London, UK). The data will be pooled as the mean difference or standardized mean difference with 95% confidence intervals for continuous outcomes and as a risk ratio with 95% confidence intervals for dichotomous outcomes. Heterogeneity between the studies in terms of the effect measures will be assessed using both the chi-squared test and the I-squared statistic. We will consider I-squared values greater than 50% and 75% indicative of substantial and high heterogeneity, respectively. In the meta-analyses, a random effects model will be used when the heterogeneity is significant (an I-squared value ≥50%), while a fixed effects model will be used when the heterogeneity is non-significant. A fixed effects model will also be used if the number of studies included in the meta-analysis is too small, which would result in poor accuracy for the inter-study variance estimates.^[15]^ In addition, we will attempt to find the reason for any significant heterogeneity, such as through subgroup analysis according to the treatment period. To identify the robustness of the meta-analysis result, we will perform sensitivity analyses by determining the effects of excluding

(1) studies with high risks of bias,
(2) studies with missing data, and
(3) outliers.

If more than 10 studies are included in the meta-analysis, we will use a funnel plot to assess publication bias.

#### 2.7.2 NMA

A random effects NMA based on the frequentist method will be carried out using Stata/MP software version 16 (StataCorp LLC, TX). Before the NMA, we will attempt to assess the clinical similarity and transitivity between studies to ensure the studies are sufficiently similar to provide valid indirect inferences. A network map will be generated to visualize the numbers and interrelations of the included interventions. The design-by-treatment interaction model will be used to detect inconsistencies and any inconsistencies between the direct and indirect evidence will be assessed using the node-splitting method. For the above-mentioned outcome measures, the intervention results will be ranked based on their surface under the cumulative ranking curve to identify the best treatment. We will present the raw data for the effect size through a network league table. Potential publication bias will be assessed using a net funnel plot if a sufficient number of studies is included. When reporting bias is implied by funnel plot asymmetry, we will attempt to determine the possible reasons.

## 3 Discussion

As effective standard therapies for pediatric ISS are lacking and GH treatment is controversial,^[4,5]^ various EATM treatments such as acupuncture, herbal medicine, and chuna, which have been used for thousands of years, are currently employed for the treatment of pediatric ISS patients in clinical settings. However, clinical studies that directly compare the effectiveness of EATMs are rare. Therefore, while evaluating the available evidence regarding the efficacy and safety of EATMs through a systematic review and pair-wise meta-analysis, we will attempt to directly and indirectly compare the various EATM interventions using NMA to obtain comprehensive evidence. In addition, since there is no systematic review comparing EATMs and GH, we will validate the evidence regarding the efficacy and safety of EATMs compared to GH. Furthermore, we will rank the interventions with respect to their effectiveness and safety for the treatment of pediatric ISS.

We believe that the results of our study will help clinicians optimize treatment protocols for ISS and will provide pediatric ISS patients a wide range of evidence-based EATM options. In addition, the evidence derived from the results of this study will be used as basic data for policy makers to enhance EATM coverage and reduce the disease burden of ISS in public health settings.

## Author contributions

**Conceptualization:** Boram Lee.

**Funding acquisition:** Boram Lee.

**Methodology:** Boram Lee, Chan-Young Kwon.

**Supervision:** Boram Lee.

**Writing – original draft:** Boram Lee.

**Writing – review & editing:** Chan-Young Kwon, Soobin Jang.
